# Radiomics based targeted radiotherapy planning (Rad-TRaP): a computational framework for prostate cancer treatment planning with MRI

**DOI:** 10.1186/s13014-016-0718-3

**Published:** 2016-11-10

**Authors:** Rakesh Shiradkar, Tarun K Podder, Ahmad Algohary, Satish Viswanath, Rodney J. Ellis, Anant Madabhushi

**Affiliations:** 1 Department of Biomedical Engineering, Case Western Reserve University, Cleveland, 44106 USA; 2 Department of Radiation Oncology, Case School of Medicine, Cleveland, 44106 USA

**Keywords:** Treatment planning, Computer aided diagnosis (CAD), Radiomics, prostate cancer

## Abstract

**Background:**

Radiomics or computer – extracted texture features have been shown to achieve superior performance than multiparametric MRI (mpMRI) signal intensities alone in targeting prostate cancer (PCa) lesions. Radiomics along with deformable co-registration tools can be used to develop a framework to generate targeted focal radiotherapy treatment plans.

**Methods:**

The Rad-TRaP framework comprises three distinct modules. Firstly, a module for radiomics based detection of PCa lesions on mpMRI via a feature enabled machine learning classifier. The second module comprises a multi-modal deformable co-registration scheme to map tissue, organ, and delineated target volumes from MRI onto CT. Finally, the third module involves generation of a radiomics based dose plan on MRI for brachytherapy and on CT for EBRT using the target delineations transferred from the MRI to the CT.

**Results:**

Rad-TRaP framework was evaluated using a retrospective cohort of 23 patient studies from two different institutions. 11 patients from the first institution were used to train a radiomics classifier, which was used to detect tumor regions in 12 patients from the second institution. The ground truth cancer delineations for training the machine learning classifier were made by an experienced radiation oncologist using mpMRI, knowledge of biopsy location and radiology reports. The detected tumor regions were used to generate treatment plans for brachytherapy using mpMRI, and tumor regions mapped from MRI to CT to generate corresponding treatment plans for EBRT. For each of EBRT and brachytherapy, 3 dose plans were generated - whole gland homogeneous ($\mathbb {P}^{\text {WH}}$) which is the current clinical standard, radiomics based focal ($\mathbb {P}^{\text {RF}}$), and whole gland with a radiomics based focal boost ($\mathbb {P}^{\text {WF}}$). Comparison of $\mathbb {P}^{\text {RF}}$ against conventional $\mathbb {P}^{\text {WH}}$ revealed that targeted focal brachytherapy would result in a marked reduction in dosage to the OARs while ensuring that the prescribed dose is delivered to the lesions. $\mathbb {P}^{\text {WF}}$ resulted in only a marginal increase in dosage to the OARs compared to $\mathbb {P}^{\text {WH}}$. A similar trend was observed in case of EBRT with $\mathbb {P}^{\text {RF}}$ and $\mathbb {P}^{\text {WF}}$ compared to $\mathbb {P}^{\text {WH}}$.

**Conclusions:**

A radiotherapy planning framework to generate targeted focal treatment plans has been presented. The focal treatment plans generated using the framework showed reduction in dosage to the organs at risk and a boosted dose delivered to the cancerous lesions.

## Introduction

Radiation therapy (RT) is one of the principal treatment modalities for localized prostate cancer and involves delivering ionizing radiation dose to the prostate, in order to destroy malignant cells. Although the specific treatment option for prostate cancer depends on the stage and grade of the tumor, RT is reported to be the most common treatment modality in patients aged 65 to 74 years, and is the second common treatment option after radical prostatectomy in younger patients [1]. Primarily, there are two main types of radiation therapy used in the treatment of prostate cancer - external beam radiation therapy (EBRT) and brachytherapy.

Prostate cancer patients are typically classified into different risk categories based on the prostate specific antigen (PSA) level, Gleason Score (GS) and T stage (tumor size) as low- (PSA ≤ 10 ng/ml, GS ≤ 6, T1 - T2a), intermediate- (10 ng/ml < PSA ≤ 20 ng/ml, GS = 7, T2b) and high-risk (PSA > 20 ng/ml, GS ≥ 8, T2c - T3a). In current clinical practice, low-risk PCa patients who are potential candidates for active surveillance but who then choose to opt out, usually undergo radical whole gland radiation therapy to ensure no cancer lesions are missed, often resulting in radiation being delivered to the surrounding healthy tissues. This typically results in significant short-term and long-term side effects including incontinence (in 5–20 % patients), sexual dysfunction (30–70 %) and bowel toxicity (5–10 %) [2, 3]. Focal therapies work by delivering a boosted radiation dose to the cancer lesion, since prostate cancer is usually focal and localized [4]. Recent studies have shown that prostate cancer focal therapy [5, 6] would result in dose minimization to the bladder and rectum while focusing therapy to the prostatic lesions, mitigating unintended side effects and treatment complications.

On the other hand, patients categorized as intermediate and high risk have a greater chance of disease progression and recurrence and are typically prescribed aggressive treatments. While enhancing dosage to the whole gland would adversely affect the surrounding healthy structures, however, introducing a focal boost to the index lesions while delivering the prescribed dose to the whole gland could potentially lead to a reduced risk of disease progression and recurrence. Therefore, focal radiotherapy, by itself or in conjunction with whole gland radiotherapy, can help in achieving better outcomes from prostate cancer radiotherapy, while also resulting in minimum radiation exposure to the adjoining structures.

Prior to therapy, a treatment plan (for either brachytherapy or EBRT) is generated which includes delineation of the prostate and surrounding structures and allocation of prescribed dosage. The prescribed dosage for brachytherapy and EBRT are pre-established numeric values according to the guidelines set aside by the Radiation Therapy Oncology Group (RTOG) [7]. EBRT planning requires these delineations to be made on a pre-treatment CT scan in order to define a planning target volume (PTV) which accounts for the radiation dose constraints, spatial margins and target dose coverage. While CT is excellent for visualizing bone structures, it does not offer very good soft tissue detail that would ensure accurate organ and tissue delineation. By comparison, MRI provides excellent soft tissue detail and contrast [8] and is being widely used for localizing PCa lesions. The PCa delineations on MRI can be transferred to CT [9, 10] to assist in EBRT planning via multi-modal registration.

In order to generate focal treatment plans, firstly an accurate spatial delineation of cancer lesions is required. Multi-parametric MRI has been shown to significantly improve the accuracy for localization and staging of prostate cancer as established by several research studies [8, 9, 11]. Nonetheless, inter-observer variability in interpreting prostate mpMRI and existence of benign confounders on imaging [12, 13] still limit accurate detection and diagnosis of CaP. Recent studies [11, 14–16] have shown radiomics based classifiers can improve the accuracy and reproducibility in localizing prostate cancer lesions on mpMRI (which includes T2w, diffusion weighted (DWI) and dynamic contrast enhanced (DCE)).

Computer-extracted texture features or radiomic features attempt to quantitatively characterize the appearance of cancer regions to better localize cancer on MRI. These radiomic features include, but not limited to, gradient based filter responses, co-occurrence features, Gabor wavelet filter based features, Law’s energy descriptors. A brief description of these features is provided in Table 2 in the “Methods” section. These features have been shown in previous studies [11, 17, 18] to characterize the appearance of prostate cancer in-vivo. Radiomics based classification of cancer involves training a machine learning classifier with the computer-extracted texture features which quantify the appearance of disease. This machine learning classifier is then used to obtain a spatial prediction map of cancer presence. The choice of imaging modality, on which a treatment plan is developed, depends on the therapy being planned. A brachtherapy treatment plan can be generated using imaging modalities such as MRI, Ultrasound imaging, CT, however, MRI offers relatively higher soft tissue detail. The treatment plans for EBRT are typically generated using CT.

The additional challenge for EBRT planning over and above brachytherapy is the transference of the detected cancer delineations from mpMRI to CT [19, 20]. The prostate gland undergoes considerable deformation on account of bladder and rectal filling, position of the patient during imaging sessions, and the shape of the surface on which the patient is resting during the scan. Therefore, a simple rigid registration may be less than optimal for multimodal MRI-CT fusion. A number of methods for deformable-registration of MRI and CT [21–24] have been developed for accurate registration of prostate between MRI and CT. Some of these approaches included semi-automatic registration methods [25, 26] which typically optimize the mutual information between the fixed and moving images, spatially aligning the prostate across the imaging modalities in order to obtain a voxel-wise correspondence. However, these methods are typically developed to align the large field of view (FOV) MRI with the corresponding CT scan. The transference of cancer delineations from the smaller FOV MRI to the corresponding CT requires a large FOV MRI as an intermediary. Therefore, a deformable registration of the small, large FOV MRI and CT would ensure an accurate transfer of cancer delineations.

Once the tumor boundaries are available on the imaging modality (MRI or CT), the next challenge involves generation of a focal treatment plan, which could potentially result in reduced genito-urinary side effects [4, 5, 27]. Banerjee et al. [28] have shown that a significant reduction in dosage to the organs at risk (OARs) was achieved using high-dose-rate focal brachytherapy, compared to whole gland and hemi-gland treatment. However, none of the previous studies on prostate cancer focal therapy included radiomics based detection of cancer lesions for therapy planning.

### Overview of Rad-TRaP

A recent review on MRI guided prostate radiotherapy [29] strongly emphasizes the role of MRI in target dose escalation for improved outcomes and recommends future integration of MRI scanners with radiation therapy delivery machines. In this paper, we present a radiomics based decision support framework for radiation treatment planning (Rad-TRaP) for prostate cancer, which combines radiomics based cancer identification and deformable registration methods for MRI-CT fusion. This framework has been used to generate radiomics based focal ($\mathbb {P}^{\text {RF}}$), and whole gland with a radiomics based focal boost ($\mathbb {P}^{\text {WF}}$) plans using the prediction results of the machine learning classifier. These plans are also compared with the whole gland homogeneous plan ($\mathbb {P}^{\text {WH}}$) which is the current clinical standard and involves uniform dose distribution to the entire gland.

**Fig. 1:**
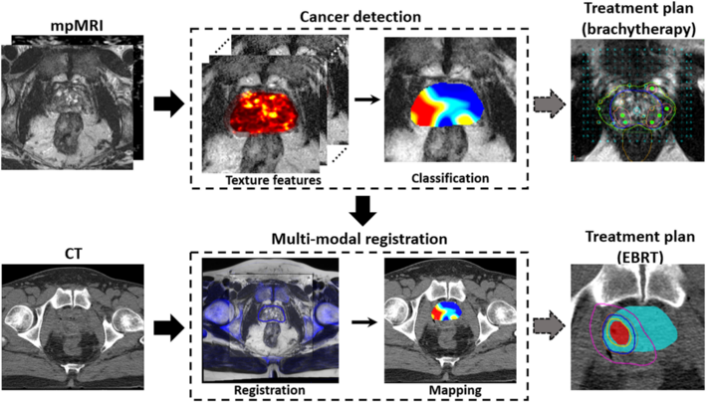
An overview of the presented framework for radiomics assisted targeted treatment radiotherapy planning (Rad-TRaP) of prostate cancer. Rad-TRaP consists of three modules - 1) voxel-wise cancer detection on MRI based on radiomic feature analysis, 2) transference of cancer delineations to CT via deformable registration of MRI and CT, and 3) generation of targeted focal radiotherapy plans for brachytherapy and EBRT

A computational pipeline of the Rad-TRaP framework is illustrated in Fig. 1, essentially consisting of the following three modules:

1. Radiomics based detection of cancerous lesions in MRI using a texture feature enabled machine learning classifier.
2. Transference of tissue, organ and delineated target volumes from MRI on to CT via multi-modal deformable co-registration.
3. Generation of focal and whole gland with focal boost treatment plans based on radiomics-detected lesions for brachytherapy and EBRT.

The specific insights gained in this study are, a) validation of the radiomics classifier for PCa detection using multi-site data, b) transference of detected PCa regions from the small FOV MRI to CT via a large FOV MRI using deformable registration and c) integration of computational tools for PCa detection and transference of these detected PCa regions across modalities to achieve targeted focal radiotherapy planning.

The rest of this paper is organized as follows. The retrospective datasets used and the pre-processing processing steps are described in the “Methods” section. This is followed by a description of the radiomic feature analysis and the building of a machine learning classifier module, a multi-modal deformable registration module and a dose plan generation module. The “Experimental design” subsection describes evaluation of each of these modules. The subsequent section presents the experimental results followed by a discussion of the observations and directions for future work. The concluding remarks are discussed in the Conclusions Section.

## Methods

### Data description

This study utilized data collected retrospectively from 2 different institutions (see Table 1 for a summary of the data description). The dataset *D*_1_ was collected from University Hospitals Cleveland and comprised 12 patients (aged 60 – 75 years) with biopsy confirmed low to intermediate risk prostate cancer (median Gleason score, 7; range, 6–8) and who had been scheduled to undergo radiation therapy. All patients underwent a 3 Tesla(T) mpMRI scan (including T2w, diffusion weighted (DWI) and dynamic contrast enhanced sequences (DCE)) prior to treatment followed by a brachytherapy seed implantation. A CT scan was acquired after the seed implantation. One of the 12 patients underwent EBRT followed by brachytherapy and so had two CT scans : one scan after EBRT and the second scan after brachytherapy. There were two T2w MRI scans acquired, one with a large field of view (FOV) and the other with a small FOV. The large FOV planning MRI contains anatomical landmarks useful for co-registration with CT whereas the small FOV diagnostic MRI has high resolution details of the prostate anatomy useful for identifying cancer.

**Table 1:** A description of the two retrospective imaging datasets employed in this work

Dataset	Number of patients	Imaging	Resolution (pixels)	Slice thickness (mm.)
*D*_1_	12	T2w (small FOV)	192×192 - 256×256	2.0-3.5
	12	T2w (large FOV)	320×320 - 512×512	3.0-5.0
	12	DWI	192×192 - 256×256	2.0-3.5
	12	CT (post-brachytherapy)	512×512	3.0
	1	CT (pre-EBRT)	512×512	3.0
*D*_2_	11	T2w	256×256 - 512×512	3.0-3.3
	11	DWI	256×256 - 512×512	3.0-3.3

*D*_2_ was used in the training of radiomics based machine learning classifier. This classifier was used to obtain cancer delineations for *D*_1_

On all the MRI scans, annotations of the cancer volume, prostate capsule and the peripheral zone were obtained, using the 3D Slicer™software, from the radiation oncologist who based them on the radiology and biopsy reports. For all future reference in this paper, the term ‘annotation’ implies delineation of contours (lesion, prostate capsule, prostate zones) on imaging. While registration with ex vivo whole-mount prostate histology specimens would provide an ideal ground truth, the patients used in this study underwent radiation therapy and therefore cancer annotations from the radiation oncologists was used. Of the 12 patients, 2 patients were excluded from the cohort: one who had cancer lesion in central gland and another who had artifacts resulting from use of an endo-rectal coil. The 10 patient studies from *D*_1_ had tumors only in the peripheral zone, all imaged using a surface coil.

The dataset *D*_2_ comprised 11 patients from Alpha 3T MRI & Diagnostic Imaging Center, New York. This second dataset was used for training a machine learning classifier for predicting prostate cancer lesions on *D*_1_. The patients from *D*_2_ were of age 45 years and above with a Gleason score less than or equal to 7 (4+3 or 3+4). All of the patients had a 3 Tesla multi-parametric MRI scan using a surface coil, prior to treatment which were used for the experiment. The MRI scans were annotated for prostate cancer lesions by an experienced radiologist based on radiology and biopsy reports, using 3D Slicer™software. All patients in *D*_2_ had cancerous lesions in the peripheral zone as well.

### Pre-processing mpMRI

**Fig. 2:**
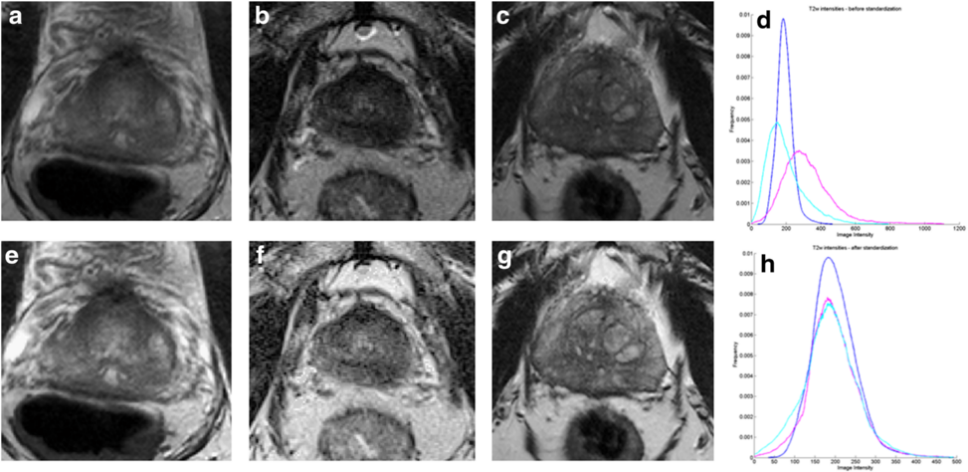
**a**, **b**, **c** show the original T2w MRI images for three patient studies from *D*_1_ and their intensity distributions within the prostate region are shown in **d**. The corresponding images after intensity standardization are shown in **e**, **f**, **g** and the standardized intensity distributions are shown in **h**

T2w MRI suffer from the issue of intensity related drift artifacts, a problem that manifests in scans acquired between patients and across scanners. These well documented [30] intensity artifacts cause the tissue specific signal intensities to vary across scans, even those obtained for the same patient on the same day and with repeat scans on the same scanner. It can be seen that the image intensity distributions, shown in Fig. 2 d, from the prostate regions on T2w MRI from different patients studies (Fig. 2 a-c) are misaligned. These varying signal distributions across scans were standardized on T2w MRI for both *D*_1_ and *D*_2_ using the method of Nyul and Udupa [31]. Briefly this approach involves computing a piece-wise linear mapping between the image intensity distributions of a template and each of the patient studies to align the distributions. This mapping is then applied to the original images to obtain standardized images (Fig. 2 e–g), in turn ensuring that the image intensity distributions are aligned across the various MRI studies (see Fig. 2 h).

### Computerized feature extraction and voxel-wise classification

Prostate cancer appears as a hypo-intense region with respect to the surrounding benign tissue on T2w MRI. This corresponds to a region with low ADC value on DWI MRI sequence. In this study, we extract radiomic features that have been previously presented as effective for automated cancer localization [18, 32, 33]. A total of 308 features are extracted (154 from T2w, 154 from ADC) on a per-voxel basis. These features are summarized in Table 2. DCE-MRI was not available for a number of studies and hence was not employed for radiomic analysis. Additionally, the role of DCE-MRI has been downplayed in the recent version of the Prostate Imaging - Reporting and Data System (PIRADS) [34]. It should also be noted that since a majority of the tumors in *D*_1_ and *D*_2_ were located in the peripheral zone, analysis was performed only in the peripheral zone; the transition zone and central gland were not considered. We do note though that the Rad-TRaP platform could certainly accommodate analysis and targeting of transitional zonal tumors as well.

**Table 2:** A brief description of the intensity and radiomic features and their significance in quantifying the appearance of prostate cancer on MRI

Feature category	Description	Significance
Signal intensity	T2w, ADC signal intensity	Regions of low signal intensities are indicative of prostate cancer lesions
First-order statistics	Mean, standard deviation, median and range; first order differentials computed using Sobel operators	Localize regions with significant intensity changes; Gradients detect edges and quantify region boundaries
Co-occurrence features	A co-occurrence matrix is a compilation of the different spatial relationships of neighboring pixel intensities. Several metrics of this matrix are computed to obtain useful features, such as Haralick features	Distinguish homogeneous regions of low intensities of cancer from hyper-intense normal regions
Gabor features	A Gabor filter is a convolution of Gaussian function with a Fourier transform at different orientations and frequencies	Gabor features quantify the appearance of cancer lesions at multiple orientations and image scales
Texture energy	Laws’ texture energy features are computed by convolution of the image with local masks that are obtained from vectors which capture local average, edge, spot, wave and ripple patterns	Texture energy quantifies the variation of pixel intensities within a fixed region of the image. Regions of image containing cancer lesions typically contain lower texture energy

Radiomic features were computed on a voxel-by-voxel basis such that every voxel **x**_*i*_ within the prostate is associated with a 308 dimensional feature vector **f**_*i*_, for all patient studies *N*,(*i*=1…*N*). Each voxel **x**_*i*_ is assigned a label **l**_*i*_ based on the expert radiologist annotation (malignant or benign).

To ensure optimal classification performance and alleviate issues related to the curse of dimensionality, the minimum redundancy maximum relevance (mRMR) feature selection scheme [35] was used to select the most discriminating features for distinguishing cancer from benign regions. The top ranked features were used to train a quadratic discriminant analysis (QDA) classifier using the cancer annotations as labels. This trained classifier generates a voxel-wise likelihood prediction **p**_*i*_ when presented with a test image as a input.The cancer likelihood prediction map is smoothed to ensure better visualization of the probabilities and remove noise. The mRMR feature selection scheme and the QDA classifier were chosen since they resulted in the best classification performance and have also been used in previous radiomics based classification studies [11, 17, 18, 32]

### Transference of detected lesions to CT via co-registration of MRI and CT

The T2w MRI is usually acquired at different fields of view (FOV), typically one at a large FOV and the other at a smaller FOV. The cancer delineations generated from the radiomcs based classifier described above are obtained on the small FOV MRI which provides much greater detail and resolution of the prostate. The registration scheme employed in this work uses the large FOV MRI as an intermediary to transfer the cancer delineations from the small FOV MRI to CT. The large FOV planning MRI contains anatomical landmarks useful for co-registration with CT, whereas the small FOV diagnostic MRI has sufficiently high anatomic detail of the prostate to enable the identification of cancer foci. The volumes of planning MRI and CT were cropped to contain only the prostate so that a meaningful registration could be executed. This approach thus mitigates the possibility of misalignment of distant internal anatomic structures that share a similar appearance. The various steps involved in the co-registration of CT and MRI are as follows.

*Step 1*: A 3D rigid registration is performed to learn a transformation **T**_*r*_ between the large FOV planning MRI $(\mathcal {M}_{p})$ and CT $(\mathcal {C})$. The large FOV MRI is required to ensure visual similarities between the two modailities such as the bone structures, muscles on MRI and CT, to drive this registration. The transformation **T**_*r*_ is computed such that it best maximizes the normalized mutual information (NMI) [36] between fixed $\mathcal {C}$ and the moving $\mathcal {M}_{p}$. This transformation **T**_*r*_ primarily brings the bone structures between these two modalities into spatial alignment. **T**_*r*_ is applied to $\mathcal {M}_{p}$ to obtain the transformed MRI ($\mathbf {T}_{r}(\mathcal {M}_{p})$). The various steps involved in the co-registration of CT and MRI are as follows.

*Step 2*: A deformable registration is performed to learn a B-spline transformation **T**_*d*_ between $\mathbf {T}_{r}(\mathcal {M}_{p})$ from the first step and the fixed $\mathcal {C}$. This transformation **T**_*d*_ accounts for the deformation of the prostate in MRI with respect to CT. It is computed by maximizing the NMI between $\mathbf {T}_{r}(\mathcal {M}_{p})$ and $\mathcal {C}$. This transformation **T**_*d*_ applied to $\mathbf {T}_{r}(\mathcal {M}_{p})$ results in the transformed MRI $\mathbf {T}_{d}(\mathbf {T}_{r}(\mathcal {M}_{p}))$. For computing **T**_*d*_, voxels only within the prostate and rectum region are used. This is to ensure that only the prostate gets deformed while other structures remain aligned from previous transformation **T**_*r*_.

The transformations **T**_*r*_ and **T**_*d*_ are applied on to the predicted cancer regions $\mathcal {P^{MR}} (\mathbf {p}_{i} \in \mathcal {P^{MR}})$ on MRI to obtain the cancer regions $\mathcal {P^{CT}}$ on CT (where $\mathbf {T}_{d}(\mathbf {T}_{r}(\mathcal {P^{MR}})) = \mathcal {P^{CT}}$). This can be seen in the Fig. 1 where the predicted cancer regions (shown as a spatial probability map) on MRI is transferred on to CT using the transformations obtained from deformable co-registration. The small and large FOV MRI are acquired at the same time point and are therefore implicitly registered. With this, the predicted cancer regions $\mathcal {P^{MR}}$ on MRI are now transferred to CT to obtain $\mathcal {P^{CT}}$.

### Dose plan generation based on predicted cancer labels

The probability scores (between 0 and 1, with 0 indicating least probability of cancer presence and 1 being the highest) assigned by the machine learning classifier are used to obtain a binary cancer volume on MRI (essentially contours showing the cancer region) at a given optimal threshold. This optimal threshold (between 0 and 1) depends on various factors including PCa grade, size of the lesion and spatial location, and therefore this threshold was decided by the radiation oncologist in this study as that at which the cancer contour best encompasses the suspicious cancer region on mpMRI. The cancer contours thus obtained will be used by the radiation oncologist using standard commercial software tools to generated whole gland homogeneous($\mathbb {P}^{\text {WH}}$), radiomics based targeted focal ($\mathbb {P}^{\text {RF}}$) and whole gland with radiomics focal boost ($\mathbb {P}^{\text {WF}}$) dose plans.

#### Implementation details for treatment planning

For brachytherapy, the treatment plans are generated on MRI using the cancer contours $\mathcal {P^{MR}}$ derived from the radiomic classifier predicted cancer probabilities. $\mathcal {P^{MR}}$ is considered as the Gross Target Volume (GTV). A focal boost was delivered to a Planning Target Volume (PTV) with a 5 mm margin surrounding the GTV to generate $\mathbb {P}^{\text {RF}}$. This margin was cropped to exclude extra-prostatic extension because the cancerous regions are confined within the prostate capsule for early stage disease (stage T1a-T2a), these are patients who are typically eligible for brachytherapy. $\mathbb {P}^{\text {WH}}$ is also generated on the same patient for comparison. Additionally, $\mathbb {P}^{\text {WF}}$ is generated for enhancing dose to GTV with a 2 mm margin. For both, $\mathbb {P}^{\text {RF}}$ and $\mathbb {P}^{\text {WF}}$, the margins for GTV were constrained to ensure that no or minimal dose spilled out of the prostate capsule or into the urethra. MIM Symphony™[37] planning tool was used for generating the radiation treatment plans for brachytherapy. The target prescription dose was 145 Gy and I-125 seeds model AgX100 (Theragenics Corporation, Buford, GA) with an Air Kerma Strength of 0.5 U being used for all the treatment plans.

In case of EBRT, the RT plans were generated after mapping the predicted cancer lesions from MRI onto CT using the multi-modal deformable registration methods. A whole gland homogeneous plan was generated with a PTV following the RTOG 0924 protocol. It is ensured that the PTV receives a prescribed dose of 79.2 Gy. A focal dose plan targeting only the cancer lesions is generated with a boosted dose of 85.8 Gy. The dose plans for EBRT were generated using the Varian Eclipse™treatment planning software [38].

### Experimental design

The modules of the Rad-TRaP framework are evaluated based on the following experiments.

#### 1. Evaluating performance of the radiomics based classifier for voxel-wise cancer prediction

The probabilities **p**_*i*_ of cancer presence generated by the machine learning classifier are evaluated against the ground truth labels **l**_*i*_ (obtained from the annotations by a radiologist) by generating the receiver operating characteristics (ROC) curve and measuring the area under the ROC curve (AUC). The ROC curve is generated by varying the probability thresholds and computing the sensitivity and specificity for each threshold in terms of the overlap with ground truth lesion.

#### 2. Evaluating multi-modal deformable registration for transference of cancer predictions

The MRI-CT registration was evaluated in terms of the Dice Similarity Co-efficient (DSC) between the prostate contours on the two modalities $\mathcal {M}_{p}$ and $\mathcal {C}$. The prostate boundary was contoured by a radiologist on each MRI and CT exam. While delineating the prostate on MRI is straightforward, the lack of structural detail on CT required the radiologist to use the appearance of brachytherapy seeds on post-treatment CT to guide the annotation.

#### 3. Evaluating treatment plans in terms of differences between dosimetric parameters

For brachytherapy, $\mathbb {P}^{\text {RF}}$ and $\mathbb {P}^{\text {WF}}$ plans are generated and compared against $\mathbb {P}^{\text {WH}}$ plan in terms of the number of seeds and needles used, target coverage and normal tissue sparing. The dosage to the rectum (*D*_1*cc*._,*D*_2*cc*_) and the bladder (*D*_2*cc*_) with the $\mathbb {P}^{\text {RF}}$ is expected to be lower compared to $\mathbb {P}^{\text {WH}}$. These parameters are expected to be similar with the $\mathbb {P}^{\text {WF}}$ compared to the $\mathbb {P}^{\text {WH}}$.

For EBRT, the dosage to the prostate, bladder, rectum, urethra, penile bulb and femoral heads are computed for each of $\mathbb {P}^{\text {RF}}$, $\mathbb {P}^{\text {WF}}$ and $\mathbb {P}^{\text {WH}}$ in terms of parameters described for brachytherapy. Also, the differences in these parameters between the $\mathbb {P}^{\text {RF}}$, $\mathbb {P}^{\text {WH}}$ and $\mathbb {P}^{\text {WF}}$, $\mathbb {P}^{\text {WH}}$ plans are computed.

## Results

### Voxel-wise cancer prediction on MRI

**Fig. 3:**
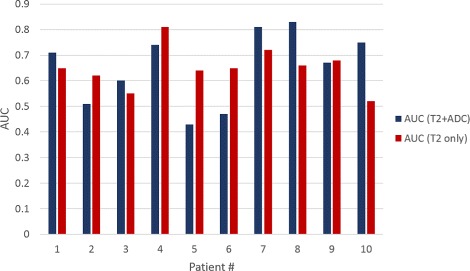
Quantitative results of the voxel-wise predictions using the radiomics trained machine learning classifier in terms of AUCs for individual patients from *D*_1_. The classifier was trained on T2w, ADC MRI sequences and T2w alone to show that misalignment between T2w and ADC MRI affects the performance of the classifier (patients 2, 5 and 6)

**Fig. 4:**
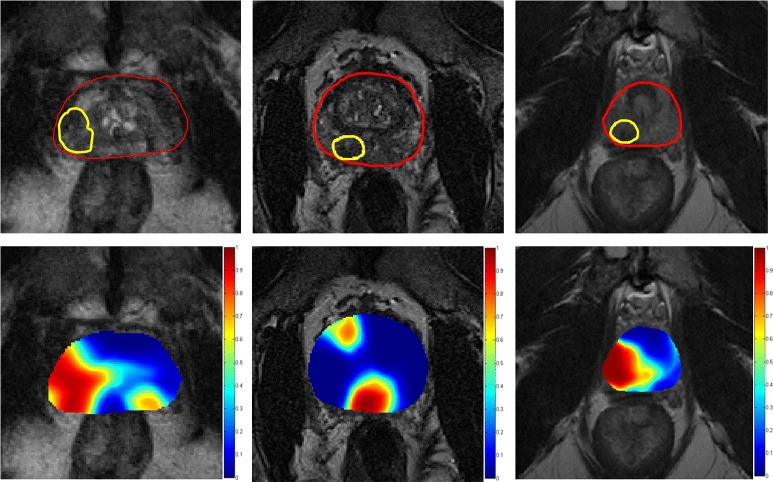
Classification results for three patients from the test set *D*_1_, obtained from the radiomics based machine learning classifier trained on *D*_2_, shown on a single representative image. The *top row* shows the T2w MRI image with the prostate capsule (*red*) and the ground truth lesion (*yellow*). The *bottom row* shows the probability maps obtained from the classifier overlaid on the image; the colorbar indicates the range of probabilities of cancer presence with *red* being the highest and *blue* being the lowest

The top performing radiomic features obtained from the feature selection scheme for *D*_2_ are summarized in Table 3. These features have also been reported by previous studies [32, 33] to be effective in detecting prostate cancer lesions on mpMRI. These features were then used to train a machine learning classifier (QDA) to identify probability of cancer presence. All predictions and evaluations were done on a voxel-by-voxel basis. This classifier was then used to obtain voxel-wise cancer predictions for *D*_1_. The AUC’s for each patient are listed in Fig. 3. A representative result of the classifier probability map is shown in Fig. 4. The regions with high probability of cancer presence are shown to be in agreement with the cancer annotations obtained from an expert radiologist.

**Table 3:** The 11 top performing features obtained from the feature selection scheme from the learning set *D*_2_ that included 3T T2w, ADC MRI sequences

#	mpMRI sequence	feature
1	T2w	Signal intensity
2	T2w	Standard deviation of signal intensity
3	T2w	Sobel gradient (*x*)
4	T2w	Haralick correlation
5	T2w	Laws energy (kernel = R5E5)
6	ADC	Signal intensity
7	ADC	Haralick energy
8	ADC	Haralick correlation
9	ADC	Haralick differential entropy
10	ADC	Laws energy (kernel = L5W5)
11	ADC	Laws energy (kernel = W5L5)

### Transference of radiomics detected lesions via co-registration of MRI and CT

**Fig. 5:**
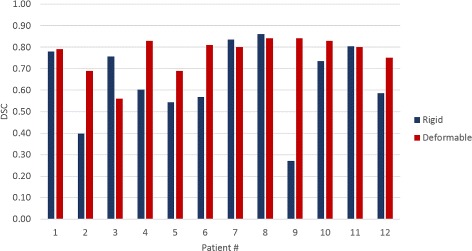
Dice similarity coefficients (DSC) evaluating the co-registration of T2w MRI and CT on *D*_1_. The DSCs from deformable registration are typically higher than those from rigid registration

**Fig. 6:**
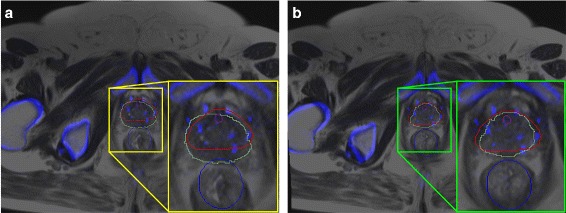
Qualitative results for multi-modal registration **a** rigid registration, **b** deformable registration. The high contrast structures from CT (*blue shade*) are overlaid onto MRI. It can be observed that the bone structures are aligned with a rigid registration alone. A deformable registration around the prostate region helps align the prostate (*red contour* on CT and *green contour* on MRI) after deformable registration. The prostate region is zoomed in and shown in the inset

The co-registration between MRI and CT was evaluated in terms of dice similarity coefficient (DSC); results are shown in Fig. 5. It should be noted that although the multi-modal registration is relevant for generating the EBRT treatment plans, the registration was evaluated for all the 12 patient studies from *D*_1_. The brachytherapy seeds could have undergone slight displacement after implantation and not all of them might lie entirely within the prostate. As reported in [39], this is not an uncommon scenario in brachytherapy seed implantation. To account for this seed displacement, DSCs were computed only on those slices containing prostate annotations both on CT and the deformed MRI. A representative result for both rigid and deformable registration is shown in Fig. 6. It can be observed that the bone structures align very closely with a rigid registration and the prostate gland requires an additional deformable registration step in order to achieve alignment.

### Treatment plan generation using the predicted cancer labels

#### 1. Brachytherapy

**Fig. 7:**
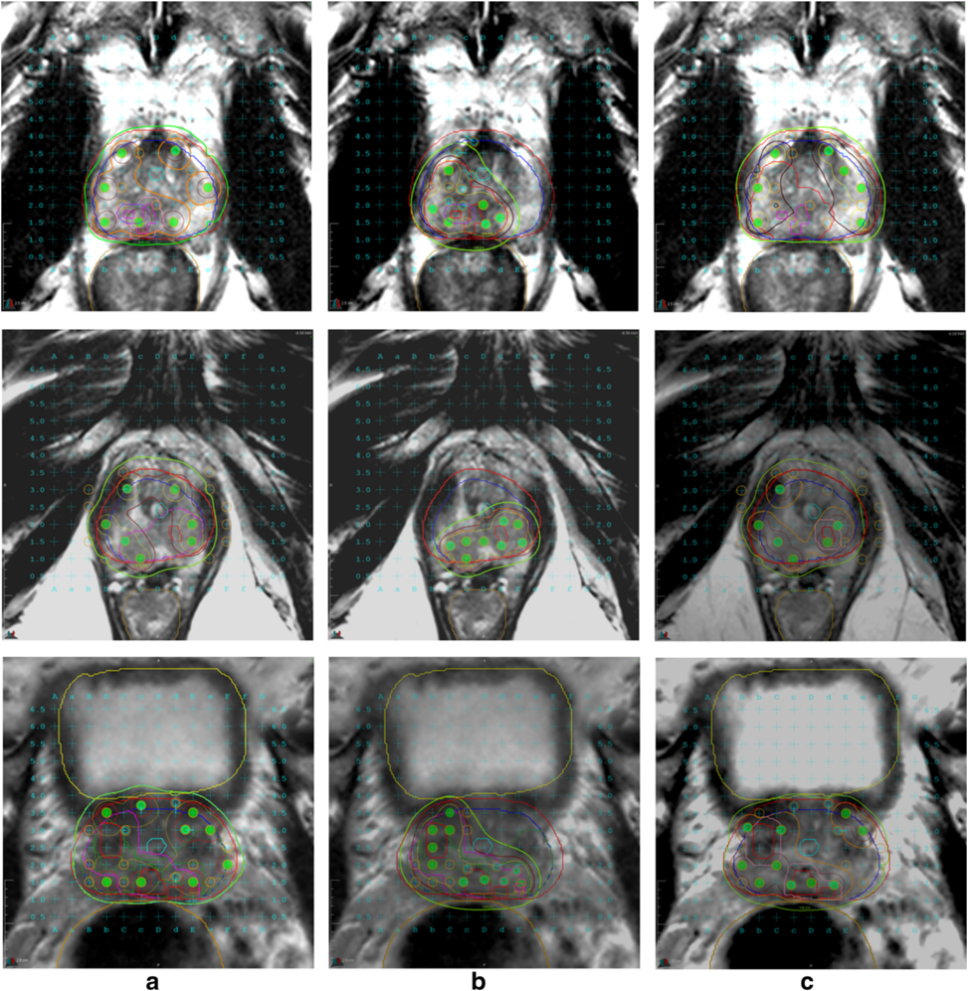
Different treatment plans for brachytherapy shown on a single slice of T2w MRI for 3 different patients (rows) - **a** $\mathbb {P}^{\text {WH}}$, **b** $\mathbb {P}^{\text {RF}}$, and **c** $\mathbb {P}^{\text {WF}}$. Plans in $\mathbb {P}^{\text {WH}}$ and $\mathbb {P}^{\text {WF}}$ cover the entire prostate (*blue contour*) and have a larger dosage (maroon colored contour shows *V*_150_) and number of needles (*green circles*) compared to $\mathbb {P}^{\text {RF}}$ in which only the cancerous region (*bright red contour* within the prostate) is covered

A representative dose plan on a single image for different patients is shown in Fig. 7 showing $\mathbb {P}^{\text {WH}}$, $\mathbb {P}^{\text {WF}}$ with a focal dose escalation to 150 *%* of prescribed dose and $\mathbb {P}^{\text {RF}}$ with a dose escalation to the dominant prostatic lesion seen on mpMRI alone. Table 4 shows the various dosimetric parameters for each of $\mathbb {P}^{\text {WH}}, \mathbb {P}^{\text {RF}}$ and $\mathbb {P}^{\text {WF}}$.

**Table 4:** A summary of the average dosimetric parameters for $\mathbb {P}^{\text {WH}}$, $\mathbb {P}^{\text {RF}}$, $\mathbb {P}^{\text {WF}}$ for brachytherapy

Parameter	Whole gland homogeneous ($\mathbb {P}^{\text {WH}}$)	Whole gland with a focal boost ($\mathbb {P}^{\text {WF}}$)	Focal ($\mathbb {P}^{\text {RF}}$)	($\mathbb {P}^{\text {WF}}-\mathbb {P}^{\text {WH}}$)	($\mathbb {P}^{\text {RF}}-\mathbb {P}^{\text {WH}}$)
Number of needles	22.00	22.10	17.20	0.10	-4.80
Number of seeds	74.80	74.20	43.00	-0.60	-31.80
Prostate					
*D*_90_ (Gy)	181.14	185.56	64.11	4.41	**-117.03**
*V*_100_ (%)	99.80	99.85	56.17	0.05	-43.63
*V*_150_ (%)	58.11	61.47	40.72	3.36	-17.38
*V*_200_ (%)	22.51	23.61	25.76	1.10	3.25
Urethra					
*D*_10_ (Gy)	192.17	195.62	182.82	3.46	**-9.34**
*V*_100_ (%)	96.55	96.62	41.38	0.07	-55.1
Rectum					
*D*_1*cc*._ (Gy)	119.81	121.77	116.79	1.96	-3.02
*D*_2*cc*._ (Gy)	102.00	102.85	92.40	0.85	**-9.60**
*V*_100_ (%)	1.14	1.41	1.63	0.27	0.49
Bladder					
*D*_2*cc*._ (Gy)	105.20	102.07	46.96	-3.13	**-58.24**
PTV					
*D*_90_ (Gy)	174.16	174.08	248.51	-0.09	**74.35**
*V*_100_ (%)	99.10	98.28	99.99	-0.82	0.90
*V*_150_ (%)	55.05	55.25	97.99	0.20	42.94
*V*_200_ (%)	21.67	21.64	76.21	-0.02	54.54

It can be observed that $\mathbb {P}^{\text {RF}}$ resulted in a better coverage of the PTV, reduction in the unneeded dosage to prostate and surrounding regions, and a marked reduction in the number of needles and seeds (highlighted in bold-face)

The dosimetric parameters *V*_100_, *V*_150_ and *V*_200_ for the prostate show a marked dose escalation to the cancer delineations, defined by the radiomic classifier, without excess dosage to the rest of the prostate. Specifically, targeted therapy with $\mathbb {P}^{\text {RF}}$ resulted in a dose escalation to the high risk regions, with a 43 *%* overall reduction in prescribed dose to the whole gland. With $\mathbb {P}^{\text {RF}}$, the PTV received a comparable dose as $\mathbb {P}^{\text {WH}}$ in terms of *V*_100_, but a significant dose escalation to the *V*_150_ and *V*_200_ at 98 *%* and 76 *%* respectively compared to $\mathbb {P}^{\text {WH}}$. More significantly, $\mathbb {P}^{\text {RF}}$ showed a marked urethral sparing which suggests reduced treatment related side effects. Also, the low 2 cc bladder volumes indicate significantly reduced dosage to the bladder. Another significant reduction is in the number of seeds and needles used. This has immediate benefits for the low-risk PCa patients who would benefit from a reduction in the number of incisions and accompanying radiation related toxicity. The dosage to the rectum, however, was observed to be comparable to $\mathbb {P}^{\text {WH}}$ due to close proximity of the rectum to the prostate, especially considering the fact that only peripheral zone lesions were considered in this study.

It can also be observed that $\mathbb {P}^{\text {WF}}$ resulted only in a marginal excess in dosage to the OARs compared to the $\mathbb {P}^{\text {WH}}$ plan. The dosage to the PTV in terms of *V*_100_, *V*_150_ and *V*_200_ is comparable to $\mathbb {P}^{\text {WH}}$. The *V*_100_ dosage from the $\mathbb {P}^{\text {WF}}$ plan to the urethra is in excess of 0.07 % and to the rectum is in excess of 0.27 % compared to $\mathbb {P}^{\text {WH}}$ showing that a whole gland treatment with focal boost helps target the lesion aggressively while minimizing damage to the surrounding structures that will result from enhancing dosage to the whole gland.

#### 2. EBRT

**Fig. 8:**
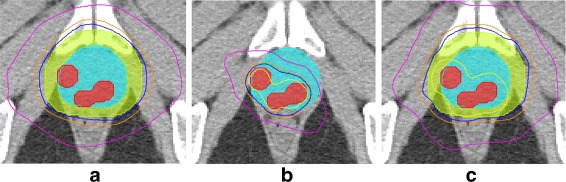
The treatment plans for EBRT shown on a single slice of T2w MRI, of a single patient - **a** $\mathbb {P}^{\text {WH}}$, **b** $\mathbb {P}^{\text {RF}}$ and **c** $\mathbb {P}^{\text {WF}}$. The red region show the cancer lesions, cyan indicates the prostate capsule. The *yellow, blue* and *magenta* contours show the radiation intensity in decreasing orders of magnitude. $\mathbb {P^{\text {RF}}}$ results in a significant reduction in dosage to extra-prostatic structures compared to $\mathbb {P}^{\text {WH}}$, and $\mathbb {P}^{\text {WF}}$ while only resulting in a marginal increase in overall dosage compared to the $\mathbb {P}^{\text {WH}}$

$\mathbb {P}^{\text {WH}}$, $\mathbb {P}^{\text {RF}}$ and $\mathbb {P}^{\text {WF}}$ are shown in Fig. 8 a, b and c respectively. The prescribed dose is shown with a green contour and the boosted dose (150 % of prescribed dose) with a dark red contour. It can be clearly noted that the radiation to the rectum and healthy prostate is significantly lower with $\mathbb {P}^{\text {RF}}$ compared to $\mathbb {P}^{\text {WH}}$. The dosimetric parameters for all the three EBRT treatment plans are tabulated in Table 5.

**Table 5:** A summary of the dosimetric parameters for $\mathbb {P}^{\text {WH}}$, $\mathbb {P}^{\text {WF}}$ and $\mathbb {P}^{\text {RF}}$ plans for a single patient

Parameter	Whole gland homogeneous ($\mathbb {P}^{\text {WH}}$)	Whole gland with focal boost ($\mathbb {P}^{\text {WF}}$)	Focal ($\mathbb {P}^{\text {RF}}$)	($\mathbb {P}^{\text {WF}}- \mathbb {P}^{\text {WH}}$)	($\mathbb {P}^{\text {RF}}-\mathbb {P}^{\text {WH}}$)
Prostate					
*V*_100 *%*_	99.6	99.32	94.21	-0.28	-5.39
*D*_98_ Gy.	80.2	79.6	85.2	-0.6	5
*D*_*min*_ (0.03 cc.) Gy.	76.7	78.5	83.8	1.8	7.1
*D*_*max*_(0.03 cc.) Gy.	81.9	102.5	91.3	20.6	9.4
Mean Dose Gy.	80.8	84.09	87.9	3.29	7.1
Bladder					
*V*_15*%*_ Gy.	60.7	65.5	1.45	4.8	**-59.25**
*V*_25*%*_ Gy.	46.9	51.7	1.85	4.8	**-45.05**
*V*_35*%*_ Gy.	36.1	42.6	1.67	6.5	**-34.43**
*V*_50*%*_ Gy.	24.1	33	1.45	8.9	**-22.65**
Rectum					
*V*_15*%*_ Gy.	63.7	60	10.5	-3.7	**-53.2**
*V*_25*%*_ Gy.	50.8	50.2	4.35	-0.6	**-46.45**
*V*_35*%*_ Gy.	36.5	42.8	2.65	6.3	**-33.85**
*V*_50*%*_ Gy.	23.2	27	1.9	3.8	**-21.3**
Penile bulb					
Mean Dose Gy.	48.98	61.97	3.65	12.99	**-45.33**
L. Femoral Head					
Mean Dose Gy.	22.1	23.3	2.3	1.2	**-19.8**
R. Femoral Head					
Mean Dose Gy.	19.8	21.3	1.4	1.5	**-18.4**

It can be observed that the $\mathbb {P}^{\text {RF}}$ resulted in significant reduction in the dosage to surrounding regions including bladder, rectum, penile bulb and the femoral heads (highlighted in bold-face)

While the PTV was adequately covered by the prescribed dose for all three treatment plans, a markedly significant reduction in the bladder, rectum, penile bulb and the femoral heads is observed with $\mathbb {P}^{\text {RF}}$. It can also be seen that $\mathbb {P}^{\text {WF}}$ resulted in a marginal increase in dosage to the surrounding structures while targeting the lesion with a boosted radiation dose.

## Discussion

In this study, a framework for generating radiomics based targeted treatment plans for focal radiation therapy of prostate cancer (Rad-TRaP) is presented. This includes 1) automatic detection of prostate cancer lesions on mpMRI using radiomic features and a machine learning classifier on a per-voxel basis, 2) transference of predicted cancer regions from mp-MRI to CT using deformable co-registration methods, and 3) a targeted focal treatment plan generation for brachytherapy and EBRT based on the predicted cancer regions.

The radiomics based classification of cancer requires the mpMRI sequences to be aligned accurately. In case of patients 2, 5 and 6, there was significant misalignment between the DWI and T2w MRI sequences that could not be resolved. This was reflected in terms of low AUC values as observed in Fig. 3. When a machine learning classifier using texture features from T2w MRI alone was trained using *D*_2_ and then tested using *D*_1_, the AUC values increased. In fact, had it not been for the gross distortion induced in the acquisition of the diffusion weighted MRI scans for these patients, the radiomics classifier would have most likely benefited from the combination of features from T2w and diffusion weighted scans. The lower AUC values from the classifier in this dataset mainly arise from the limitations of dataset, which include lower resolution of the MRI data (especially ADC sequences), smaller data size and non-availability of DCE MRI sequence.

The registration approach appears to be dependant on the type of coil (body or endorectal) used for mpMRI acquisition. It can be observed from Fig. 5 that in case of patient 3, who had an MRI scan with an endo-rectal coil, a rigid registration of MRI and CT is sufficient.

While the results obtained with the initial set of 23 patients in this study is promising, there are avenues for improvement in future work. Firstly, the diagnostic MRI used in this study for cancer detection was of lower magnetic strength; using an MRI of higher magnetic strength may result in an improvement in cancer detection performance of the radiomics based classifier. Also, additional sequences including DCE MRI, if available, could be explored for radiomics based cancer detection.

Secondly, the Rad-TRaP framework was validated on a retrospective dataset of low to intermediate risk prostate cancer patients. Experiments on a larger cohort of patients with a wider spectrum of risk categories will be important for evaluating the generalizability of the framework.

Another avenue for future work is to develop an automated method of identifying the threshold to obtain contours for the lesion from the spatial probability maps. The voxel wise predictions obtained from the radiomics classifier do not take into account factors including size of the lesion, cancer grade and spatial location of the cancer lesion. These are important factors in accurately determining the optimal threshold. In order to automatically define an optimal threshold, the radiomics classifier would have to be trained and evaluated on a much larger cohort of studies, one with additional diversity of cancers corresponding to different Gleason grades.

The Rad-TRaP framework presented in this work has been validated on patient studies with peripheral zone tumors. The framework can be easily adapted for central gland lesions by curating an appropriate dataset consisting of cancer lesions in the central gland. In fact, the framework is amenable to embedding any trained classifier. For instance, integrating a classifier trained on specific data at a specific site or scanner can be easily achieved.

## Conclusions

Rad-TRaP presents a unique decision support framework for radiation oncologists, potentially helping them generate effective and targeted treatment options for low, intermediate and high risk prostate cancer patients. A whole gland homogeneous dose plan, a whole gland dose plan with a focal boost and a targeted focal dose plan were generated based on the cancer predictions for brachytherapy and EBRT. These dose plans were evaluated and compared in terms of several dosimetric parameters, our simulation results suggesting a significant reduction in radiation dosage to the rectum and bladder while delivering prescribed dosage to the cancer lesions using a focal dose plan. The whole gland plan with a focal boost resulted in delivery of boosted dose to the target lesions without excess spillover of radiation to the surrounding extra-prostatic structures.

## Abbreviations

ADC: Apparent diffusion coefficient
CT: Computed tomography
DCE: Dynamic contrast enhanced
DWI: Diffusion weighted imaging
EBRT: External beam radiation therapy
FOV: Field of view
GTV: Gross tumor volume
mpMRI: Multi-parametric MRI
MRI: Magnetic resonance imaging
OAR: Organs at risk
PCa: Prostate cancer
PTV: Planning target volume
RT: Radiation therapy
RTOG: Radiation therapy oncology group
T: Tesla

## References

[1] Cancer Treatment and Survivorship, Facts and Figures 2014–15. http://www.cancer.org/acs/groups/content/@research/documents/document/acspc-042801.pdf. Accessed 24 Oct 2016.

[2] Sanda MG, Dunn RL, Michalski J, Sandler HM, Northouse L, Hembroff L, Lin X, Greenfield TK, Litwin MS, Saigal CS, Mahadevan A, Klein E, Kibel A, Pisters LL, Kuban D, Kaplan I, Wood D, Ciezki J, Shah N, Wei JT (2008). Quality of life and satisfaction with outcome among prostate-cancer survivors. N Engl J Med.

[3] Wilt TJ, MacDonald R, Rutks I, Shamliyan TA, Taylor BC, Kane RL (2008). Systematic review: Comparative effectiveness and harms of treatments for clinically localized prostate cancer. Ann Intern Med.

[4] Eggener SE, Scardino PT, Carroll PR, Zelefsky MJ, Sartor O, Hricak H, Wheeler TM, Fine SW, Trachtenberg J, Rubin MA, Ohori M, Kuroiwa K, Rossignol M, Abenhaim L (2007). Focal therapy for localized prostate cancer: A critical appraisal of rationale and modalities. J Urol.

[5] Karavitakis M, Ahmed HU, Abel PD, Hazell S, Winkler MH (2011). Tumor focality in prostate cancer: implications for focal therapy. Nat Rev Clin Oncol.

[6] Ahmed HU, Hindley RG, Dickinson L, Freeman A, Kirkham AP, Sahu M, Scott R, Allen C, der Meulen JV, Emberton M (2012). Focal therapy for localised unifocal and multifocal prostate cancer: a prospective development study. Lancet Oncol.

[7] Radiation Therapy Oncology Group, Clinical Trials. https://www.rtog.org/ClinicalTrials/ProtocolTable.aspx. Accessed 24 Oct 2016.

[8] Kirkham AP, Emberton M, Allen C (2006). How good is mri at detecting and characterising cancer within the prostate?. Eur Urol.

[9] Villeirs GM, De Meerleer GO (2007). Magnetic resonance imaging (mri) anatomy of the prostate and application of mri in radiotherapy planning. Eur J Radiol.

[10] Dirix P, Haustermans K, Vandecaveye V. The value of magnetic resonance imaging for radiotherapy planning. In: Seminars in Radiation Oncology. Elsevier: 2014. p. 151–9, doi:10.1016/j.semradonc.2014.02.003. 10.1016/j.semradonc.2014.02.003 24931085

[11] Lemaître G, Martí R, Freixenet J, Vilanova JC, Walker PM, Meriaudeau F (2015). Computer-aided detection and diagnosis for prostate cancer based on mono and multi-parametric mri: A review. Comput Biol Med.

[12] Hoeks CM, Hambrock T, Yakar D, Hulsbergen–van de Kaa CA, Feuth T, Witjes JA, Fütterer JJ, Barentsz JO (2013). Transition zone prostate cancer: detection and localization with 3-t multiparametric mr imaging. Radiology.

[13] Ruprecht O, Weisser P, Bodelle B, Ackermann H, Vogl TJ (2012). Mri of the prostate: interobserver agreement compared with histopathologic outcome after radical prostatectomy. Eur J Radiol.

[14] Litjens GJS, Elliott R, Shih NNC, Feldman MD, Kobus T, Hulsbergen-van de Kaa C, Barentsz JO, Huisman HJ, Madabhushi A (2016). Computer-extracted features can distinguish noncancerous confounding disease from prostatic adenocarcinoma at multiparametric mr imaging. Radiology.

[15] Niaf E, Rouvière O, Mège-Lechevallier F, Bratan F, Lartizien C (2012). Computer-aided diagnosis of prostate cancer in the peripheral zone using multiparametric mri. Phys Med Biol.

[16] Viswanath SE, Bloch BN, Chappelow J, Toth R, Rofsky NM, Genega EM, Lenkinski RE, Madabhushi A (2012). Central gland and peripheral zone prostate tumors have significantly different quantitative imaging signatures on 3 tesla endorectal, in vivo t2-weighted mr imagery. J Magn Reson Imaging.

[17] Litjens GJS, Elliott R, Shih N, Feldman M, Barentsz JO, Hulsbergen-van de Kaa CA, Kovacs I, Huisman HJ, Madabhushi A. Distinguishing Prostate Cancer from Benign Confounders Via a Cascaded Classifier on Multi-parametric MRI. In: SPIE Medical Imaging: 2014. p. 903512–0351214, doi:10.1117/12.2043751. http://dx.doi.org/10.1117/12.2043751.

[18] Ginsburg SB, Viswanath SE, Bloch BN, Rofsky NM, Genega EM, Lenkinski RE, Madabhushi A (2015). Novel pca-vip scheme for ranking mri protocols and identifying computer-extracted mri measurements associated with central gland and peripheral zone prostate tumors. J Magn Reson Imaging JMRI.

[19] Korsager AS, Carl J, Østergaard LR (2013). Mr-ct registration using a ni-ti prostate stent in image-guided radiotherapy of prostate cancer. Med Phys.

[20] Zhong H, Wen N, Gordon JJ, Elshaikh MA, Movsas B, Chetty IJ (2015). An adaptive mr-ct registration method for mri-guided prostate cancer radiotherapy. Phys Med Biol.

[21] Kessler ML (2014). Image registration and data fusion in radiation therapy. Br J Radiol.

[22] Sparks R, Nicolas Bloch B, Feleppa E, Barratt D, Moses D, Ponsky L, Madabhushi A (2015). Multiattribute probabilistic prostate elastic registration (mapper): Application to fusion of ultrasound and magnetic resonance imaging. Med Phys.

[23] Rivest-Hénault D, Dowson N, Greer PB, Fripp J, Dowling JA (2015). Robust inverse-consistent affine ct–mr registration in mri-assisted and mri-alone prostate radiation therapy. Med Image Anal.

[24] Toth R, Traughber B, Ellis R, Kurhanewicz J, Madabhushi A (2014). A domain constrained deformable (docd) model for co-registration of pre-and post-radiated prostate mri. Neurocomputing.

[25] Maes F, Collignon A, Vandermeulen D, Marchal G, Suetens P (1997). Multimodality image registration by maximization of mutual information. IEEE Trans Med Imaging.

[26] Klein S, Staring M, Pluim JP (2007). Evaluation of optimization methods for nonrigid medical image registration using mutual information and b-splines. IEEE Trans Image Process.

[27] Ahmed HU, Hindley RG, Dickinson L, Freeman A, Kirkham AP, Sahu M, Scott R, Allen C, Van der Meulen J, Emberton M (2012). Focal therapy for localised unifocal and multifocal prostate cancer: a prospective development study. Lancet Oncol.

[28] Banerjee R, Park SJ, Anderson E, Demanes DJ, Wang J, Kamrava M (2015). From whole gland to hemigland to ultra-focal high-dose-rate prostate brachytherapy: A dosimetric analysis. Brachytherapy.

[29] McPartlin A, Li X, Kershaw L, Heide U, Kerkmeijer L, Lawton C, Mahmood U, Pos F, van As N, van Herk M (2016). Mri-guided prostate adaptive radiotherapy–a systematic review. Radiother Oncol.

[30] Vovk U, Pernuš F, Likar B (2007). A review of methods for correction of intensity inhomogeneity in mri. IEEE Trans Med Imaging.

[31] Nyul LG, Udupa JK. On standardizing the mr image intensity scale. Magn Reson Med. 1999; 42(6):1072–81. doi:10.1002/(SICI)1522-2594(199912)42:6<1072::AID-MRM11>3.0.CO;2-M. 10.1002/(sici)1522-2594(199912)42:6<1072::aid-mrm11>3.0.co;2-m 10571928

[32] Viswanath SE, Bloch NB, Chappelow JC, Toth R, Rofsky NM, Genega EM, Lenkinski RE, Madabhushi A (2012). Central gland and peripheral zone prostate tumors have significantly different quantitative imaging signatures on 3 tesla endorectal, in vivo t2-weighted mr imagery. J Magn Reson Imaging.

[33] Wibmer A, Hricak H, Gondo T, Matsumoto K, Veeraraghavan H, Fehr D, Zheng J, Goldman D, Moskowitz C, Fine SW (2015). Haralick texture analysis of prostate mri: utility for differentiating non-cancerous prostate from prostate cancer and differentiating prostate cancers with different gleason scores. Eur Radiol.

[34] Weinreb JC, Barentsz JO, Choyke PL, Cornud F, Haider MA, Macura KJ, Margolis D, Schnall MD, Shtern F, Tempany CM, Thoeny HC, Verma S, Weinreb JC (2016). PI-RADS Prostate Imaging-Reporting and Data System: 2015. Version 2. Eur Urol.

[35] Peng H, Long F, Ding C (2005). Feature selection based on mutual information criteria of max-dependency, max-relevance, and min-redundancy. IEEE Trans Pattern Anal Mach Intell.

[36] Pluim JP, Maintz JA, Viergever MA (2000). Image registration by maximization of combined mutual information and gradient information. Medical Image Computing and Computer-Assisted Intervention–MICCAI 2000.

[37] MIM Software Inc, Cleveland O. MIM Symphony. http://www.mimsoftware.com/products/symphony/. Accessed 24 Oct 2016.

[38] Systems VM. Eclipse Treatment Planning System. https://www.varian.com/oncology/products/software/treatment-planning/eclipse. Accessed 24 Oct 2016.

[39] Poggi MM, Gant DA, Sewchand W, Warlick WB (2003). Marker seed migration in prostate localization. Int J Radiat Oncol Biol Phys.

